# Toward a clearer vision: epidemiology and symptom-based clinical patterns of dry eye disease in the Saudi population

**DOI:** 10.3389/fmed.2026.1763735

**Published:** 2026-02-05

**Authors:** Mariam M. AlEissa, Ahmed Mousa, Hala A. Alosimi, Rana A. Alosimi, Abrar A. Alhawsawi, Reem Alkharji, Amera S. AlQahtani, Raniah S. Alotibi, Sulaiman I. Alquwayfili, Sajjad Ahmad, Deepak P. Edward

**Affiliations:** 1 College of Medicine, Alfaisal University, Riyadh, Saudi Arabia; 2 Research Department, King Khaled Eye Specialist Hospital, Riyadh, Saudi Arabia; 3 Population Health Observatory, Ministry of Health, Riyadh, Saudi Arabia; 4 Computational Sciences Department at the Centre for Genomic Medicine (CGM), King Faisal Specialist Hospital & Research Center, Riyadh, Saudi Arabia; 5 Public Health Authority, Public Health Lab, Riyadh, Saudi Arabia; 6 King Saud Medical City, Riyadh First Health Cluster, Riyadh, Saudi Arabia; 7 College of Medicine, Princess Nourah Bent Abdulrahman University, Riyadh, Saudi Arabia; 8 Ophthalmology Division, Department of Surgery, College of Medicine, University of Jeddah, Jeddah, Saudi Arabia; 9 Research Department, Health Sciences Research Centre, Princess Nourah Bint Abdulrahman University, Riyadh, Saudi Arabia; 10 Medical Genetics, Hopital Purpan, CHU de Toulouse, Toulouse, France; 11 Department of Clinical Laboratory Sciences, College of Applied Medical Sciences, King Saud bin Abdulaziz University for Health Sciences, Riyadh, Saudi Arabia; 12 King Abdullah International Medical Research Center, Riyadh, Saudi Arabia; 13 Ministry of National Guard–Health Affairs, Riyadh, Saudi Arabia; 14 Lean Business Services, Riyadh, Saudi Arabia; 15 Institute of Ophthalmology, University College of London, London, United Kingdom; 16 Moorfields Eye Hospital, London, United Kingdom; 17 Moorfields/UCL NIHR Biomedical Research Centre, London, United Kingdom

**Keywords:** contact lenses, digital device use, dry eye disease, epidemiology, public health, Saudi Arabia

## Abstract

**Background:**

Dry Eye Disease (DED) is a multifactorial ocular surface disorder characterized by tear film instability and inflammation, leading to ocular discomfort, irritation, and visual disturbance. In Saudi Arabia, environmental conditions, ergonomic, and behavioral factors such as digital device use and contact lens wear may exacerbate DED symptoms. However, large-scale population-based studies examining the epidemiological and clinical determinants of DED remain limited.

**Methods:**

This cross-sectional study was conducted using a validated online questionnaire distributed across Saudi Arabia. The survey comprised four domains: sociodemographic characteristics, awareness and knowledge of eye health, behavioral and environmental risk factors, and clinical symptoms related to DED and contact lens use. Data from 1,009 participants were analyzed using SPSS version 29. Potential associations between DED domains and demographic or behavioral variables were examined using chi-square and t-tests, with significance set at *p* < 0.05.

**Results:**

Among the 1,009 respondents, females represented 56.7%, and the majority were aged 35–44 years (29.3%). The mean domain scores indicated that “Irritation/Dryness” was the most affected domain (47.5 ± 22.8). The *“Symptoms domain”* was significantly associated with age, gender, marital status, education, and occupation (*p* < 0.001), whereas the *“Activity Limitations domain”* significantly correlated with age, marital status, education, and occupation (*p* < 0.05). Both *“Eye Discomfort”* and *“Irritation/Dryness”* domains were significantly related to gender (*p* < 0.001) and lifestyle factors, particularly hours spent on phones and computers (*p* < 0.001). Prolonged digital exposure and female gender were the strongest predictors of increased DED symptom burden.

**Conclusion:**

Dry Eye Disease is a prevalent and multifactorial health concern among the Saudi population, strongly influenced by demographic, behavioral, ergonomic, and environmental determinants. Women and younger adults with high exposure to digital devices are particularly vulnerable. Implementing public health interventions, including awareness programs, workplace ergonomics, and AI-based digital screening, may be crucial to reducing the disease burden and advancing Vision 2030 goals for preventive eye health.

## 1 Introduction

Dry eye disease (DED) is a common, multifactorial disorder worldwide that affects the ocular surface, caused by inflammation and tear film instability, characterized by visual disturbances, irritation, and ocular discomfort (1, 2). Inconsistency of the methods used for diagnostic criteria can play a crucial role in the varying prevalence rate (3, 4). The global prevalence varies depending on the diagnostic criteria, and climate estimates range from 5 to 50% (5). In Saudi Arabia, a study reported a prevalence of DED of approximately 74.9% across all severity levels, with only 30.4% classified as severe cases (6). Another study indicated a prevalence of 18.2% among medical students, highlighting that even within specific demographics, the rates remain significant (7). Further, the prevalence is higher in women than in men, influenced by autoimmune predisposition, hormonal changes, and the use of contact lenses (8, 9).

In Saudi Arabia, the arid climate, combined with environmental and behavioral factors, significantly contributes to the occurrence of DED due to chronic exposure to dust, wind, heat, and high rates of tear evaporation, which can be exacerbated by indoor air conditioning or outdoor travel (6, 10). Further DED among young adults is linked to Digital device use by prolonged screen exposure, and low blink rates (11). Contact lenses also play a crucial role in exacerbating ocular inflammation, particularly in extended-wear lenses (12, 13). Number of local studies link diseases like thyroid dysfunction, diabetes, and autoimmune disorders, or medications antidepressants and antihistamines, to DED (14–16). Moreover, lifestyle plays a critical role in exacerbating disease severity, influenced by factors such as poor diet, smoking, and dehydration (17–19). This study investigated the pathophysiology and epidemiology of DED in Saudi Arabia, with a focus on the impact of contact lenses and screening different age groups that are most affected by DED, as well as the health issues that may contribute to DED. In addition, this study suggests public health measures that prioritize education, early intervention, and routine monitoring to prevent DED and promote community eye health in general, with a special focus on women.

## 2 Method

This study received ethical clearance from the Ministry of Health, Riyadh, Kingdom of Saudi Arabia, through the National Committee of Bioethics (NCBE-KACST, H-01-R-009) and was approved under IRB log number 25-18E.

A cross-sectional (online) survey, found in Supplementary Table 1, was distributed via a Google form for six months, which was administered starting 11 February 2025. All the patients/participants’ legal guardians/next of kin provided signed informed consent to participate in this study. The survey was created based on professional opinions, earlier suggestions, and research inquiries. First, the survey was developed in English and then translated into Arabic by bilingual translators. It was subsequently evaluated by multilingual translators and approved by the study team and external participants to ensure the broader community would understand it. Outside specialists reviewed the survey to ensure that its language and content were accurate. Cronbach’s alpha test was used to assess the survey’s reliability and consistency, yielding a value of 0.73 for each item. Participants were informed that the survey would take 10 to 15 min to complete. The primary method employed in our study to select participants was snowball sampling technique. The online Arabic version of the survey was distributed through two social media platforms, WhatsApp and Shahim from the Ministry of Health to avoid duplicating or repeated submissions, just one contribution per IP address was authorized.

The online survey examined four main domains: (1) Sociodemographic characteristics (e.g., age, education, province, income, and employment status), (2) Awareness and knowledge of eye health and DED, (3) Behavioral, ergonomic and environmental factors (e.g., screen use, contact lens habits, smoking, and exposure to dust or air conditioning), and (4) Self-reported signs, symptoms and experiences related to DED and contact lens use. The questionnaire was created in English and then translated into Arabic, the local language, by fluent speakers of both languages, and reviewed to ensure that it is appropriate for a broad audience.

We calculated the minimum sample size needed for our investigation using the Slovin method (*n* = *N*/(1 + Ne2)) (20). Using an approximate starting population of 35,000,000 inhabitants according to the Saudi General Authority for Statistics (21), and a 0.5 margin of error (d), we determined that 400 was the required sample size to develop concrete evidence.

## 3 Statistical analysis

The responses to the questionnaire were analyzed in five domains: the *“sociodemographic domain,”* the *“Symptoms Score domain”* (Questions 1–5), the *“Activity Limitations domain”* (Questions 6–9) which reflects inability to perform some activities due to DES, the *“Eye Discomfort domain”* (Questions 10–12), and the *“Irritation and Dryness domain”* (Q25, Q27, Q30, Q31, Q33, Q35). Findings from each of those domains were transformed into a score according to the associated Likert scale of answers, where a scale between (0–100) was developed, where 0 = None of the time, and 100 = All the time.

Data from the questionnaires were then exported to Microsoft Excel 365 (Microsoft Corporation, Redmond, Washington, United States). The analysis was conducted using SPSS version 29.0 (IBM Inc., Chicago, Illinois, United States). Descriptive analysis was done, where categorical variables were presented in the form of frequencies and percentages [No. (%)], while continuous variables were presented as mean (±SD), range [min – max]. The inferential analysis was conducted using the Chi-squared test for associations between categorical variables and to link the sociodemographic characteristics to the outcome of interest *(Fisher’s exact test was used whenever indicated)*. Meanwhile, the independent Student’s t-test was used to compare the mean difference in Likert score domains across continuous variables *(Mann–Whitney U test was alternatively used, whenever indicated)*. The confidence interval level was set to 95% where a corresponding *p*-value threshold was identified as 0.05, whereas any output *p* below 0.05 would be interpreted as an indicator of statistical significance.

## 4 Result

A total of 1,009 participants took part in this study, spanning a broad age range from 18 to over 65 years, with all major age groups fairly well represented. The largest group was those aged 35–44 (N = 295, 29.3%). Females accounted for 56.7% (n = 572) of participants, while males made up 43.1% (n = 433), and 64.4% (n = 649) were married. Most respondents had a bachelor’s degree (452, 44.8%) and worked full-time (551, 54.7%). Details of the sociodemographic characteristics of the recruited subjects are presented in Table 1.

**Table 1:** Sociodemographic characteristics of the recruited subjects.

Characteristic	Category	No. (%)	*p*-value
Age group	[<18]	6 (0.6)	<0.001
	[18–24]	135 (13.4)	
	[25–34]	181 (18)	
	[35–44]	295 (29.3)	
	[45–54]	184 (18.3)	
	[55–64]	143 (14.2)	
	[≥65]	64 (6.3)	
Gender	Male	433 (43.1)	<0.001
	Female	572 (56.7)	
Marital status	Single	290 (28.8)	<0.001
	Married	649 (64.4)	
	Divorced	49 (4.9)	
	Widowed	19 (1.9)	
Education	Less than high school	21 (2.1)	<0.001
	High school diploma or equivalent	114 (11.3)	
	Some college	79 (7.8)	
	Bachelor’s degree	452 (44.8)	
	Master’s degree	169 (16.8)	
	Doctorate or professional degree	173 (17.2)	
Occupation	Employed full-time	551 (54.7)	<0.001
	Employed part-time	35 (3.5)	
	Unemployed and looking for work	78 (7.7)	
	Unemployed and not looking for work	59 (5.8)	
	Student	132 (15.1)	
	Retired	152 (15.1)	

**Figure 1:**
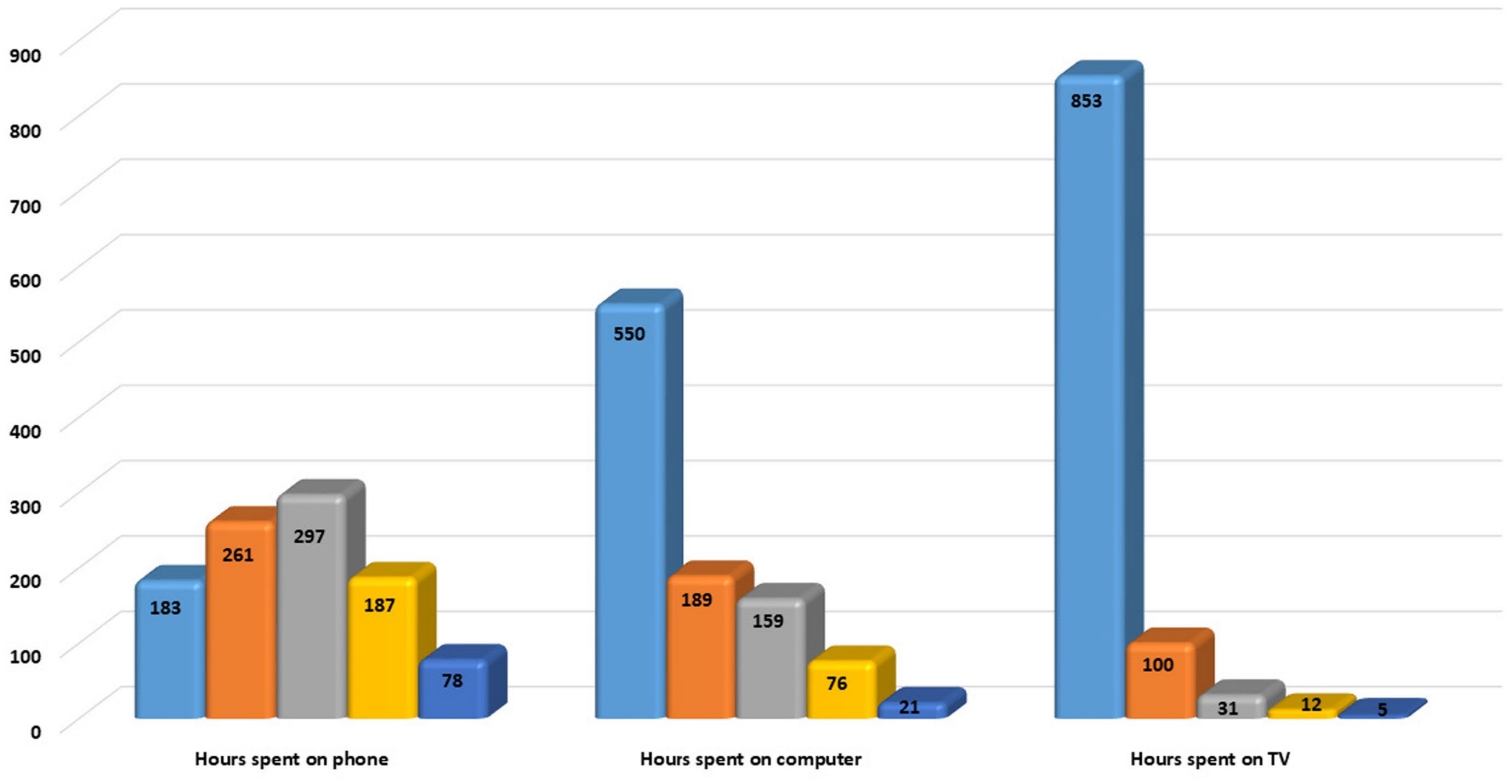
A diagram represents hours spent on the phone, computer, and television. Navy blue represents 1–3 h, orange represents 3–5 h, grey represents 5–7 h, yellow represents 7–10 h, and dark blue represents 10–13 h.

The analysis of the average range of hours spent on electronic devices and watching TV revealed that 297 (29.5%) spent 5–7 h on cell phones, 550 (55.3%) spent 1–3 h on a computer, and 853 (85.2%) spent 1–3 h watching TV. Details of the frequency of hours spent on electronic devices are illustrated in Figure 1.

**Figure 2:**
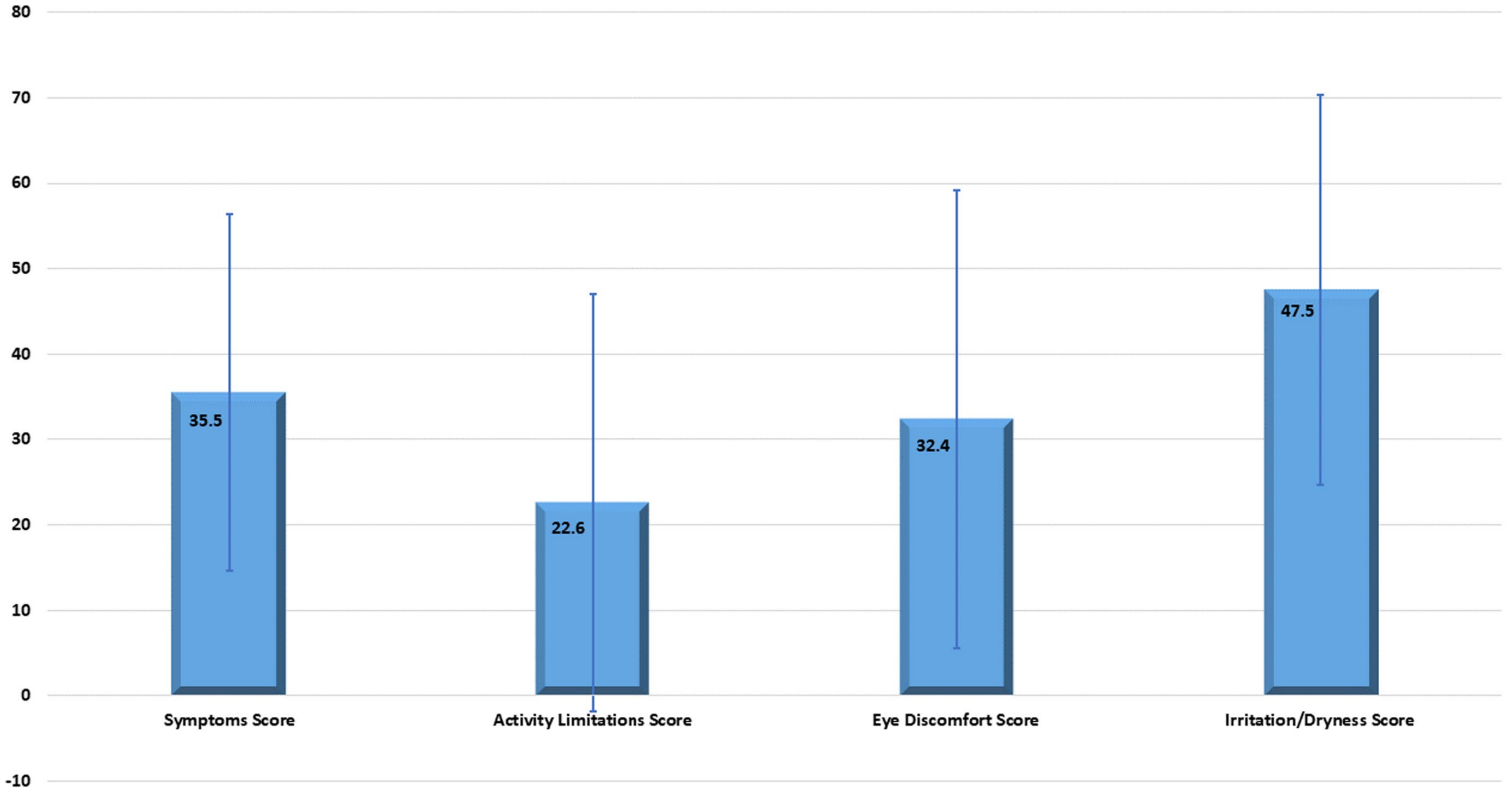
Whiskers plot presents the average domain score of aggregated questions.

Analysis of the data from the DED questionnaire revealed that almost all average domain scores were below 50%, except for the Irritation/Dryness score, which was nearly at the midpoint, 47.5 (±22.8). Details of average domain scores are demonstrated in Figure 2. The range of scores among the domains was wide.

Examining the association between different study domains and sociodemographic characteristics revealed distinct patterns. The *“Symptoms domain,”* representing the overall frequency and intensity of DED manifestations such as blurred vision, eye fatigue, and a sense of ocular heaviness, showed a highly significant association with age (youngest), gender (female), marital status (widowed), education (lowest level), and occupation (student & part time employees) (*p* < 0.001 for all). In contrast, the *“Activity Limitations domain,”* which measures the functional impact of DED on daily activities such as reading, driving, or screen use, was significantly associated with age (youngest) (*p* < 0.001), marital status (widowed) (*p* = 0.01), education (lowest level) (*p* = 0.01), and occupation (retired) (*p* = 0.009), but not with gender (*p* = 0.095).

The *“Eye Discomfort domain,”* focusing on sensations of pain, burning, or stinging, demonstrated strong associations with age (youngest) and gender (female) (*p* < 0.001 for both), and significant relationships with marital status (widowed) and education (postgraduate) (*p* = 0.007 for both), though not with occupation. Meanwhile, the *“Irritation/Dryness domain,”* which captures perceptions of ocular dryness, grittiness, and itching related to tear film instability, was highly associated with gender (female) (*p* < 0.001) and significantly related to marital status (widowed), education (lowest level), and occupation (part time employee) (*p* = 0.017, 0.017, and 0.002, respectively) (Table 2). As the DES is highly associated with outdoor activities, we conducted a comparative analysis among those who are working outdoors and those who are working indoors using the occupation characteristics by aggregating the outdoors and indoors categories. The analysis revealed that there was a significantly significant difference across both categories in both the “Symptoms domain” (average score 37.6 (21.7), and 30.1 (17.8), *p* < 0.001), and the “Eye Discomfort domain” “(average score 33.9 (27.6), and 28.9 (24.3), *p* = 0.005), for outdoor and indoor groups, respectively, while there was no association in both the “Activity Limitations domain and “Irritation/Dryness domain,” *p* = 0.620 and 0.745 for both domains, respectively.

**Table 2:** Association between average domain scores and demographic characteristics.

Characteristic	Categories	Domain							
		Symptoms		Activity Limitations		Eye Discomfort		Irritation/Dryness	
		Score Mean (SD)	*p*-value	Score Mean (SD)	*p*-value	Score Mean (SD)	*p*-value	Score Mean (SD)	*p*-value
Age	[<18]	50.8 (38.9)	<0.001	38.5 (48.2)	<0.001	45.8 (45.3)	<0.001	47.2 (17.4)	0.175
	[18–24]	39.6 (23.8)		17.9 (23.9)		33.2 (29.1)		48.2 (21.5)	
	[25–34]	37.8 (21.8)		20.9 (25.4)		39.6 (30.4)		50.4 (25.6)	
	[35–44]	35.9 (20.2)		17.7 (21.6)		32.1 (25.7)		46.9 (21.7)	
	[45–54]	35.7 (21.4)		29.7 (25.5)		30.5 (25.3)		48.3 (23.9)	
	[55–64]	31.1 (16.7)		29.4 (23.9)		28.5 (23.1)		46.5 (22.6)	
	[≥65]	26.2 (15.5)		23.3 (21.4)		25.7 (21.3)		40.9 (18.7)	
Gender	Male	30.4 (18.5)	<0.001	21.1 (23.3)	0.095	27.3 (24.1)	<0.001	39.2 (17.1)	<0.001
	Female	39.4 (21.8)		23.7 (25.2)		36.3 (28.1)		53.8 (24.5)	
Marital status	Single	38.7 (22.8)	<0.001	18.7 (23.9)	0.01	35.5 (29.2)	0.007	47.5 (21.2)	0.017
	Married	33.4 (19.7)		23.9 (24.4)		30.4 (25.4)		46.7 (23.2)	
	Divorced	40.8 (21.1)		25.9 (24.3)		39.1 (28.5)		56.2 (26.8)	
	Widowed	42.6 (21.7)		28.9 (26.2)		39.5 (26.0)		55.0 (17.4)	
								
Education	Less than high school	40.7 (26.6)	<0.001	30.9 (28.9)	0.01	31.3 (30.6)	0.007	52.2 (29.7)	0.017
	High school diploma or equivalent	36.2 (24.5)		20.1 (22.5)		30.6 (26.9)		48.9 (23.7)	
	Some college	35.3 (20.9)		21.1 (24.2)		34.9 (27.1)		48.5 (23.3)	
	Bachelor’s degree	35.4 (20.3)		22.2 (25.1)		32.5 (27.4)		47.4 (22.4)	
	Master’s degree	37.4 (21.0)		22.6 (22.8)		35.6 (27.1)		47.3 (22.1)	
	Doctorate or professional degree	32.8 (18.9)		25.1 (24.7)		29.5 (24)		46.1 (22.8)	
Occupation	Employed full-time	36.8 (20.9)	<0.001	22.7 (24.4)	0.009	33.8 (27.3)	0.13	46.5 (21.9)	0.002
	Employed part-time	40.4 (20.1)		24.5 (20.5)		33.1 (24.6)		61.5 (26.4)	
	Unemployed and looking for work	32.5 (20.2)		15.1 (20.7)		31.6 (27.0)		48.5 (24.6)	
	Unemployed and not looking for work	30.3 (18.2)		22.1 (22.4)		26.3 (23.0)		51.2 (24.3)	
	Student	40.5 (28.8)		20.5 (26.7)		34.6 (29.9)		48.9 (22.9)	
	Retired	28.9 (16.2)		27.8 (24.8)		28.6 (23.4)		44.9 (22.1)	

Although all four domains assess different aspects of DED, the *“Symptoms domain”* represents a broad composite measure of general disease burden, whereas the *“Eye Discomfort”* and *“Irritation/Dryness”* domains provide granular insight into specific sensory experiences of the condition. On the other hand, investigating the association between different study domains and the hours spent on the phone, computer, and watching TV programs revealed a statistically significant association between the *“Symptoms domain.”* This association was significant for both variables, hours spent on the phone and hours spent on the computer (*p* < 0.001). Meanwhile, a significant association was detected between the *“Symptoms domain”* and the number of hours spent watching TV (*p = 0.011*). In terms of the *“Activity limitations domain,”* there was a significant association with the hours spent on the phone and on TV (*p = 0.044* and 0.002, respectively, for telephone and TV hours). Meanwhile, there was no association between hours spent on the computer and the *“Activity limitations domain”* (*p = 0.058*). As regards the *“Eye discomfort domain,”* there was an association with hours spent on phone (*p* = 0.016), and hours spent on computer (*p* < 0.001), while the association between such domain and the hours spent on TV was insignificant (*p* = 0.307). The *“Irritation/Dryness domain”* was found to be highly associated with the hours spent on the phone (*p* < 0.001) and was significantly associated with both the hours spent on the computer (*p = 0.003*) and the hours spent on TV (*p = 0.038*) (Table 3).

**Table 3:** Association between average domain scores and hours spent on phone, computer, and TV.

Characteristic	Categories	Domain							
		Symptoms		Activity limitations		Eye discomfort		Irritation/dryness	
		Score Mean (SD)	*p*-value	Score Mean (SD)	*p*-value	Score Mean (SD)	*p*-value	Score Mean (SD)	*p*-value
Hours spent on the phone	[1–3]	31.6 (20.9)	<0.001	24.6 (25.2)	0.044	29.6 (27.4)	0.016	43.3 (21.6)	<0.001
	[3–5]	34.1 (20.3)		22.9 (25.8)		30.3 (25.1)		44.9 (21.8)	
	[5–7]	34.1 (18.4)		19.1 (21.8)		32.6 (26.0)		47.9 (20.7)	
	[7–10]	39.5 (22.4)		24.8 (24.2)		34.9 (28.7)		49.9 (24.1)	
	[10–13]	45.4 (24.0)		25.0 (26.4)		40.4 (27.8)		59.2 (28.3)	
Hours spent on computer	[1–3]	32.6 (20.4)	<0.001	20.7 (22.8)	0.058	29.7 (25.7)	<0.001	46.6 (22.7)	0.003
	[3–5]	37.6 (20.7)		25.6 (25.9)		32.2 (27.0)		46.9 (21.9)	
	[5–7]	37.9 (20.3)		24.2 (26.2)		38.2 (27.5)		47.2 (21.2)	
	[7–10]	42.2 (20.5)		25.4 (26.4)		37.5 (28.3)		55.5 (24.9)	
	[10–13]	54.0 (26.2)		28.3 (28.8)		46.4 (32.1)		59.5 (30.7)	
Hours spent on TV	[1–3]			21.7 (23.9)	0.002				
	[3–5]			29.1 (26.5)					
	[5–7]			20.9 (21.1)					
	[7–10]	34.8 (20.7)	0.011	26.6 (30.6)		31.8 (26.7)	0.307	46.9 (22.3)	0.038
	[10–13]	39.6 (21.1)		55.0 (37.3)		36.2 (27.3)		52.2 (24.3)	

## 5 Discussion

The distribution of ocular diseases varies across populations due to demographic, behavioral, and environmental determinants (22). In this national cross-sectional study, we provide a comprehensive epidemiological assessment of dry eye disease (DED) in the Saudi population, demonstrating significant associations with gender, age, digital device use, occupation, and lifestyle factors.

### 5.1 Comparison with Saudi prevalence studies

Previous Saudi studies have reported substantial variability in DED prevalence. Alrabghi et al. (6) documented a prevalence of 74.9% across all severity levels, whereas Aljammaz et al. (7) reported a prevalence of 18.2% among medical students. Although our study did not estimate prevalence using diagnostic criteria, the observed symptom burden was considerable, particularly among females and young adults. These discrepancies can be attributed to methodological differences, including variations in sampled populations and assessment tools. Specifically, Alrabghi et al. included both pediatric and adult participants and applied diagnostic definitions, whereas our study employed a multi-domain system, which assessed symptom severity and functional impact across multiple domains. Similarly, Aljammaz et al. focused exclusively on medical students and utilized the McMonnies questionnaire, which assesses fewer symptom dimensions. Such differences likely explain the variability in reported prevalence across Saudi studies.

### 5.2 Gender-related differences

Consistent with previous Saudi reports (6, 7), females constituted the majority of our cohort (56.7%) and exhibited significantly higher scores in the Eye Discomfort and Irritation/Dryness domains (*p* < 0.001). However, the magnitude of symptom severity observed in our study was greater, particularly among middle-aged women (35–54 years). This may reflect our broader age distribution and occupational diversity. Biological mechanisms, including autoimmune predisposition and hormonal fluctuations, likely contribute to this disparity (8, 9). Moreover, postmenopausal hormonal decline has been shown to impair meibomian gland function and tear film stability (1), further exacerbating symptoms.

### 5.3 Age patterns and digital exposure

DED symptoms and activity limitations were significantly associated with age (*p* < 0.001), peaking among young adults (18–34 years). This pattern aligns with global observations reported by Britten-Jones et al. (3), who described a U-shaped age distribution. In our cohort, younger participants reported the highest digital device usage, which demonstrated the strongest association across all symptom domains (*p* < 0.001). These findings corroborate Al-Mohtaseb et al. (11), who demonstrated that prolonged screen exposure reduces blink rates and increases tear evaporation. The stronger association observed in our study may be explained by the high proportion of students and full-time employees in our sample.

### 5.4 Environmental exposure and occupational factors

Environmental determinants have been emphasized in Saudi studies as major contributors to DED. Alrabghi et al. (6) and Al-Dossary (10) identified hot climate, dust exposure, and air-conditioning as dominant risk factors. While our results confirmed significantly higher symptom scores among outdoor workers compared to indoor workers (*p* < 0.001), environmental exposure ranked secondary to digital device use. This discrepancy likely reflects regional and occupational differences between studies. Prior investigations were conducted in climate-specific regions with high outdoor exposure, whereas our nationally representative sample included a larger proportion of indoor digital workers. These findings suggest a shift in dominant risk factors toward behavioral determinants in contemporary Saudi society.

### 5.5 Marital status, education, and employment

Divorced and widowed participants exhibited significantly higher Irritation/Dryness scores (*p* = 0.017). This association has not been consistently reported in previous Saudi literature and may be linked to psychosocial stress and medication use, such as antidepressants, known to impair tear production (14). Additionally, lower educational level and full-time employment were associated with increased symptom burden, likely due to prolonged screen exposure and reduced awareness of preventive eye care. While similar occupational trends have been reported globally (3), few Saudi studies have explored these sociodemographic determinants, highlighting the novelty of our findings.

### 5.6 Lifestyle factors

Lifestyle behaviors, including dehydration and smoking, were associated with symptom exacerbation, consistent with findings by Alsahly et al. (17) and Tariq et al. (18). However, in our cohort, these factors ranked below digital exposure and gender, indicating that modern behavioral patterns exert a stronger influence on DED burden in Saudi Arabia.

### 5.7 Risk factor ranking and public health implications

Based on the magnitude and consistency of associations observed in our analysis, risk factors were ranked according to their statistical strength (Table 4). Prolonged digital device use emerged as the primary risk factor, followed by female gender and younger age. Environmental exposure through outdoor occupations ranked fourth, while sociodemographic factors such as marital status, employment, and education showed moderate associations. Lifestyle factors demonstrated weaker but consistent effects.

**Table 4:** Ranking of risk factors for dry eye disease (DED) based on study findings.

Rank	Risk factor	Evidence from this study	Statistical significance	Key affected domains
1	Prolonged digital device use	Strong positive association with increasing hours of use	p < 0.001	All domains
2	Female gender	Higher mean symptom scores	p < 0.001	Eye Discomfort, Irritation/Dryness
3	Younger age (18–34 years)	Highest symptom burden	p < 0.001	Symptoms, Activity limitation
4	Outdoor occupation	Higher symptom scores than indoor workers	p < 0.001	Symptoms, Eye discomfort
5	Marital status (divorced/widowed)	Higher dryness and irritation scores	p = 0.017	Irritation/Dryness
6	Employment status	Full-time workers & students more affected	p < 0.001	Symptoms
7	Educational level	Lower education associated with higher severity	*p* < 0.01	All domains
8	Lifestyle factors	Smoking & dehydration worsen symptoms	Consistent trend	Symptoms

This ranking highlights a transition toward modifiable behavioral risks, underscoring the need for targeted preventive strategies. Integrating digital screening tools and educational programs within primary healthcare, universities, and workplaces may facilitate early detection and intervention, aligning with Vision 2030 objectives.

The current study may have some potential limitations, such as building on subjective reporting of the respondents on some clinical-related symptoms and manifestations, as well as the potentiality of sampling bias due to conducting an online survey. However, we methodologically believe that the large enough sample size has minimised such bias via reflecting the community in terms of sociodemographic characteristics and hence the normative data. The used strategy followed a rigid sampling technique that was approved by the Ministry of Health for large sample size studies. Thus, the increased sample size should have reached the suitability threshold of precision that would enable generalisation of the findings to the community. Meanwhile, reporting such a large data set with an increased number of questions may have limited the capacity of conduct and report detailed associations and go for multivariate analysis based on the four domains’ scores as an outcome of interest. Further reports will tackle such issue focusing on specific research points.

### 5.8 Recommendation

These findings highlight the need for targeted, evidence-based recommendations derived directly from the identified risk factors in this study. Given the strong association between prolonged digital device use and increased DED symptom burden across all domains (*p* < 0.001), screening strategies should be implemented within primary healthcare, schools, and occupational health settings, particularly for students and full-time employees.

Furthermore, as female gender and younger age groups demonstrated significantly higher symptom severity (*p* < 0.001), awareness campaigns should prioritize women and digital workers, emphasizing regular screening, contact-lens hygiene, and adequate hydration.

Importantly, the significantly higher symptom and eye discomfort scores observed among outdoor workers compared to indoor workers (*p* < 0.001) justify evidence-informed workplace policies, including ergonomic screen practices for indoor workers and protective measures for outdoor workers, such as eye protection and regulation of airflow and humidity. While these environmental interventions were not directly measured, they represent practical public health responses to the risk factors identified in this study.

Finally, given the association between DED symptoms and reported systemic conditions, ocular surface assessment should be integrated into chronic disease management protocols, particularly for patients with diabetes, thyroid disorders, and autoimmune diseases.

## 6 Conclusion

The study confirms that in Saudi Arabia, DED is a multifactorial health problem with high prevalence impacted by gender, digital exposure, and environmental stressors. DED is significantly associated with demographics, where essential preventive strategies and public health education play a crucial role. Incorporating frameworks for national DED screening for workers and chronic disease will improve the quality of life, reduce morbidity, and advance Vision 2030’s goals of population-wide health promotion and early disease prevention.

## Data Availability

The raw data supporting the conclusions of this article will be made available by the authors, without undue reservation.
