# Correction: Toward a clearer vision: epidemiology and symptom-based clinical patterns of dry eye disease in the Saudi population

**DOI:** 10.3389/fmed.2026.1821004

**Published:** 2026-03-17

**Authors:** Mariam M. AlEissa, Ahmed Mousa, Hala A. Alosimi, Rana A. Alosimi, Abrar A. Alhawsawi, Reem Alkharji, Amera S. AlQahtani, Raniah S. Alotibi, Sulaiman I. Alquwayfili, Sajjad Ahmad1, Deepak P. Edward

**Affiliations:** 1 College of Medicine, Alfaisal University, Riyadh, Saudi Arabia; 2 Research Department, King Khaled Eye Specialist Hospital, Riyadh, Saudi Arabia; 3 Population Health Observatory, Ministry of Health, Riyadh, Saudi Arabia; 4 Computational Sciences Department at the Centre for Genomic Medicine (CGM), King Faisal Specialist Hospital & Research Center, Riyadh, Saudi Arabia; 5 Public Health Authority, Public Health Lab, Riyadh, Saudi Arabia; 6 King Saud Medical City, Riyadh First Health Cluster, Riyadh, Saudi Arabia; 7 College of Medicine, Princess Nourah Bent Abdulrahman University, Riyadh, Saudi Arabia; 8 Ophthalmology Division, Department of Surgery, College of Medicine, University of Jeddah, Jeddah, Saudi Arabia; 9 Research Department, Health Sciences Research Centre, Princess Nourah Bint Abdulrahman University, Riyadh, Saudi Arabia; 10 Medical Genetics, Hopital Purpan, CHU de Toulouse, Toulouse, France; 11 Department of Clinical Laboratory Sciences, College of Applied Medical Sciences, King Saud bin Abdulaziz University for Health Sciences, Riyadh, Saudi Arabia; 12 King Abdullah International Medical Research Center, Riyadh, Saudi Arabia; 13 Ministry of National Guard–Health Affairs, Riyadh, Saudi Arabia; 14 Lean Business Services, Riyadh, Saudi Arabia; 15 Institute of Ophthalmology, University College of London, London, United Kingdom; 16 Moorfields Eye Hospital, London, United Kingdom; 17 Moorfields/UCL NIHR Biomedical Research Centre, London, United Kingdom

**Keywords:** contact lenses, digital device use, dry eye disease, epidemiology, public health, Saudi Arabia

Affiliation “College of Medicine, Alfaisal University, Riyadh, Saudi Arabia” was erroneously listed as affiliation 2 in the original article. This affiliation has now been changed to affiliation 1, and affiliation “Research Department, King Khaled Eye Specialist Hospital, Riyadh, Saudi Arabia” has been changed to affiliation 2.

The original version of this article has been updated.

