# Supplementary material for: Toward a clearer vision: epidemiology and symptom-based clinical patterns of dry eye disease in the Saudi population
Source: Front Med (Lausanne). 2026 Feb 5;13:1763735. doi: 10.3389/fmed.2026.1763735 (PMC12927477; doi:10.3389/fmed.2026.1763735)
Supplement: Supplementary file 1 [file Data_Sheet_1.docx]

**Supplementary 1:** The questionnaire used in this study

| Dry eye disease in the Saudi Arabia population | جفاف العين لدى سكان المملكة العربية السعودية |
| --- | --- |
| The research aims to study the epidemiology, pathophysiology, and age-related features of dry eye disease (DED) in the Saudi population, particularly those wearing contact lenses. A comprehensive public health strategy will include age-specific conditions, health promotion programs, and preventive techniques. A questionnaire related to these topics will be used to reduce the negative effects on human health.  By participating in this survey, your answers will be treated with strict confidentiality and minimal risk, whether physical, professional, financial, emotional, or social. So we hope that you will participate and answer all the questions  *Please note that all data is confidential and will only be used for scientific study purposes.  If you have any questions about the research, please contact  Principal Investigator Dr. Mariam AlEissa.  Medical Genetics Consultant at the Public Health Authority  Associate Professor at Alfaisal University  With our sincere thanks and appreciation,  Research Team | يهدف هذا المشروع البحثي على دراسة مسحية استقصائيه والفيزيولوجيا المرضية والسمات المرتبطة بالعمر لمرض جفاف العين (DED) لدى الشعب السعودي عامه وبشكل خاص مرتدو العدسات اللاصقة. وسيتم تغطية استراتيجية شاملة للصحة العامة، تشمل الحالات المرضيه لعمر معين، وبرامج تعزيز الصحة، والتقنيات الوقائية. ومن أجل الحد من الآثار السلبية على صحة الانسان، من خلال الاستبيانات المرتبطة بهذه المواضيع.  من خلال مشاركتك بهذا الاستبيان سيتم التعامل مع الإجابات بسرية تامة مع الحد الأدنى من المخاطر، سواء كانت جسدية ومهنية، ومالية وعاطفية واجتماعية. لذا نأمل المشاركة والاجابه على جميع الأسئلة  * يرجى ملاحظة أن جميع البيانات سرية ولن تستخدم إلا لأغراض الدراسة العلمية  إذا كان لديك أي أسئلة حول البحث، يرجى التواصل مع  الباحث الرئيسي د مريم بنت محمد العيسى. استشاري وراثة طبيه هيئة الصحة العامة  استاذ مشارك متعاون في جامعة الفيصل  مع خالص شكرنا وتقديرنا،  فريق البحث |
| **Demographic Questions** | **الأسئلة الديموغرافية** |
| 1. **What is your age?**    - 18-24    - 25-34    - 35-44    - 45-54    - 55-64    - 65 or older 2. **What is your gender?**    - Male    - Female 3. **What is your marital status?**    - Single    - Married    - Divorced    - Widowed 4. **What is the highest level of education you have completed?**    - Less than high school    - High school diploma or equivalent    - Some college    - Bachelor’s degree    - Master’s degree    - Doctorate or professional degree 5. **What is your employment status?**     - Employed full-time    - Employed part-time    - Unemployed and looking for work    - Unemployed and not looking for work    - Student    - Retired 6. How long do you spend of your time in front of  - Phone   1,2,3,4,5,6,7,8,9,10,11,12   - TV   1,2,3,4,5,6,7,8,9,10,11,12   - Computer   1,2,3,4,5,6,7,8,9,10,11,12 | 1. **كم عمرك؟**    - 18-24    - 25-34    - 35-44    - 45-54    - 55-64    - 65 أو أكثر 2. **ماهو جنسك؟**  - ذكر - أنثى  1. **ما هي حالتك الاجتماعية؟**    - أعزب    - متزوج    - مطلق    - أرمل 2. **ما هو أعلى مستوى تعليمي أنهيته؟**    - أقل من الثانوية العامة    - شهادة الثانوية العامة أو ما يعادلها    - بعض الدراسات الجامعية    - درجة البكالوريوس    - درجة الماجستير    - درجة الدكتوراه أو شهادة مهنية 3. **ما هي حالتك الوظيفية؟**    - موظف بدوام كامل    - موظف بدوام جزئي    - عاطل عن العمل ويبحث عن وظيفة    - عاطل عن العمل ولا يبحث عن وظيفة    - طالب    - متقاعد 4. كم المدة التي تقضيها يوميا  - الهاتف   1,2,3,4,5,6,7,8,9,10,11,12   - التلفاز   1,2,3,4,5,6,7,8,9,10,11,12   - الكمبيوتر   1,2,3,4,5,6,7,8,9,10,11,12 |
| **Section 1: Symptoms**   1. Have your eyes been sensitive to light?    - 4: All of the time    - 3: Most of the time    - 2: Half of the time    - 1: Some of the time    - 0: None of the time 2. Have your eyes felt gritty?    - 4: All of the time    - 3: Most of the time    - 2: Half of the time    - 1: Some of the time    - 0: None of the time 3. Have your eyes felt painful or sore?    - 4: All of the time    - 3: Most of the time    - 2: Half of the time    - 1: Some of the time    - 0: None of the time 4. Have you experienced blurred vision?    - 4: All of the time    - 3: Most of the time    - 2: Half of the time    - 1: Some of the time    - 0: None of the time 5. Have you experienced poor vision?    - 4: All of the time    - 3: Most of the time    - 2: Half of the time    - 1: Some of the time    - 0: None of the time | **القسم الأول: الأعراض**   1. **هل شعرت بحساسية في عينيك تجاه الضوء؟**    - 4: طوال الوقت    - 3: معظم الوقت    - 2: نصف الوقت    - 1: أحيانًا    - 0: أبدًا 2. **هل شعرت بجفاف و بخشونة في عينيك؟**    - 4: طوال الوقت    - 3: معظم الوقت    - 2: نصف الوقت    - 1: أحيانًا    - 0: أبدًا 3. **هل شعرت بألم أو وجع في عينيك؟**    - 4: طوال الوقت    - 3: معظم الوقت    - 2: نصف الوقت    - 1: أحيانًا    - 0: أبدًا 4. **هل واجهت رؤية ضبابية؟**    - 4: طوال الوقت    - 3: معظم الوقت    - 2: نصف الوقت    - 1: أحيانًا    - 0: أبدًا 5. **هل واجهت ضعفًا في الرؤية؟**    - 4: طوال الوقت    - 3: معظم الوقت    - 2: نصف الوقت    - 1: أحيانًا    - 0: أبدًا |
| **Section 2: Limitations in Activities**   1. Has reading been difficult due to your eyes?    - 4: All of the time    - 3: Most of the time    - 2: Half of the time    - 1: Some of the time    - 0: None of the time    - N/A: Not applicable 2. Has driving at night been difficult due to your eyes?    - 4: All of the time    - 3: Most of the time    - 2: Half of the time    - 1: Some of the time    - 0: None of the time    - N/A: Not applicable 3. Has working with a computer or using an ATM been difficult due to your eyes?    - 4: All of the time    - 3: Most of the time    - 2: Half of the time    - 1: Some of the time    - 0: None of the time    - N/A: Not applicable 4. Has watching TV been difficult due to your eyes?    - 4: All of the time    - 3: Most of the time    - 2: Half of the time    - 1: Some of the time    - 0: None of the time    - N/A: Not applicable | **القسم الثاني: القيود في الأنشطة**   1. **هل كان من الصعب القراءة بسبب عينيك؟**    - 4: طوال الوقت    - 3: معظم الوقت    - 2: نصف الوقت    - 1: أحيانًا    - 0: أبدًا    - غير مطبق 2. **هل كان من الصعب القيادة ليلاً بسبب عينيك؟**    - 4: طوال الوقت    - 3: معظم الوقت    - 2: نصف الوقت    - 1: أحيانًا    - 0: أبدًا    - غير مطبق 3. **هل كان من الصعب استخدام الكمبيوتر أو أجهزة الصراف الآلي بسبب عينيك؟**    - 4: طوال الوقت    - 3: معظم الوقت    - 2: نصف الوقت    - 1: أحيانًا    - 0: أبدًا    - غير مطبق 4. **هل كان من الصعب مشاهدة التلفاز بسبب عينيك؟**    - 4: طوال الوقت    - 3: معظم الوقت    - 2: نصف الوقت    - 1: أحيانًا    - 0: أبدًا    - غير مطبق |
| **Section 3: Discomfort in Specific Situations**   1. Have your eyes felt uncomfortable in windy conditions?    - 4: All of the time    - 3: Most of the time    - 2: Half of the time    - 1: Some of the time    - 0: None of the time 2. Have your eyes felt uncomfortable in areas with low humidity (e.g., very dry conditions)?    - 4: All of the time    - 3: Most of the time    - 2: Half of the time    - 1: Some of the time    - 0: None of the time 3. Have your eyes felt uncomfortable in air-conditioned areas?    - 4: All of the time    - 3: Most of the time    - 2: Half of the time    - 1: Some of the time    - 0: None of the time 4. **Have you ever had drops prescribed or other treatment for dry eye?**    - Yes (2)    - No (0)    - Uncertain (1) 5. **Do you ever experience any of the following symptoms?** *(Please underline those that apply.)*    - Soreness (1)    - Scratchiness (1)    - Dryness (1)    - Grittiness (1)    - Burning (1) 6. **How often do your eyes have these symptoms?**    - Never (0)    - Sometimes (1)    - Often (2)    - Constantly (3) 7. **Do you regard your eyes as being unusually sensitive to cigarette smoke, smog, air conditioning, or central heating?**    - Yes (2)    - No (0)    - Sometimes (1) 8. **Do your eyes become very red and irritated when swimming in chlorinated fresh water?**    - Not applicable    - Yes (2)    - No (0)    - Sometimes (1) 9. **Are your eyes dry and irritated the day after drinking alcohol?**    - Not applicable    - Yes (2)    - No (0)    - Sometimes (1) 10. **Please underline those that you take**:     - Antihistamine tablets (1)     - Antihistamine eye drops (1)     - Diuretics (fluid tablets) (1)     - Sleeping tablets (1)     - Tranquilizers (1)     - Oral contraceptives (1)     - Medication for duodenal ulcer (1)     - Medication for digestive problems (1)     - Medication for high blood pressure (1)     - Other: ________ (1) 11. **Do you suffer from arthritis?**     - Yes (2)     - No (0)     - Uncertain (1) 12. **Do you experience dryness of the nose, mouth, throat, chest, or vagina?**     - Never (0)     - Sometimes (1)     - Often (2)     - Constantly (3) 13. **Do you suffer from thyroid abnormality?**     - Yes (2)     - No (0)     - Uncertain (1) 14. **Are you known to sleep with your eyes partially open?**     - Yes (2)     - No (0)     - Uncertain (1) 15. **Do you have eye irritation when you wake up after sleeping?**     - Yes (2)     - No (0)     - Uncertain (1) | **الثالث: الانزعاج في مواقف معينة**   1. **هل شعرت بعدم راحة في عينيك في ظروف العواصف الجوية؟**  - 4: طوال الوقت - 3: معظم الوقت - 2: نصف الوقت - 1: أحيانًا - 0: أبدًا  1. **هل شعرت بعدم راحة في عينيك في الأماكن ذات الرطوبة المنخفضة (مثل الظروف الجافة جدًا)؟**  - 4: طوال الوقت - 3: معظم الوقت - 2: نصف الوقت - 1: أحيانًا - 0: أبدًا  1. **هل شعرت بعدم راحة في عينيك في الأماكن المكيفة؟**  - 4: طوال الوقت - 3: معظم الوقت - 2: نصف الوقت - 1: أحيانًا - 0: أبدًا  1. **هل سبق أن وصف لك طبيب قطرات للعين أو علاج آخر لجفاف العين؟**  - 2: نعم - 1: لا - 0: غير متأكد  1. **هل تعاني من أي من الأعراض التالية؟ (يرجى تحديد تلك التي تنطبق عليك)**  - ألم (1) - شعور بالخدش (1) - جفاف (1) - شعور بالرملية (1) - حرقة (1)  1. **كم مرة تعاني عيناك من هذه الأعراض؟**  - أبدًا (0) - أحيانًا (1) - غالبًا (2) - باستمرار (3)  1. **هل تعتبر عيناك حساسة بشكل غير عادي لدخان السجائر، الضباب، التكييف، أو التدفئة المركزية؟**  - نعم (2) - لا (0) - أحيانًا (1)  1. هل تصبح عيناك حمراء جدًا ومتهيجة عند السباحة في المياه المعالجة بالكلور؟  - غير مطبق - نعم (2) - لا (0) - أحيانًا (1)  1. هل تعاني من جفاف وتهيج في العين بعد شرب الكحول؟  - غير مطبق - نعم (2) - لا (0) - أحيانًا (1)  1. **يرجى تحديد الأدوية التي تتناولها:**  - أقراص مضادة للهستامين (1) - قطرات مضادة للهستامين (1) - مدرات البول (أقراص السوائل) (1) - أقراص منومة (1) - مهدئات (1) - حبوب منع الحمل (1) - دواء لقرحة الاثني عشر (1) - دواء لمشاكل الجهاز الهضمي (1) - دواء لارتفاع ضغط الدم (1) - أدوية أخرى: ________ (1)  1. **هل تعاني من التهاب المفاصل؟**  - نعم (2) - لا (0) - غير متأكد (1)  1. **هل تعاني من جفاف في الأنف، الفم، الحلق، الصدر، أو المهبل؟**  - أبدًا (0) - أحيانًا (1) - غالبًا (2) - باستمرار (3)  1. **هل تعاني من اضطراب في الغدة الدرقية؟**  - نعم (2) - لا (0) - غير متأكد (1)  1. **هل تستطيع النوم وأنت تبقي عينيك مفتوحتين جزئيًا؟**  - نعم (2) - لا (0) - غير متأكد (1)  1. **هل تشعر بتهيج في العين عند الاستيقاظ بعد النوم؟**  - نعم (2) - لا (0) - غير متأكد (1) |
| **1. Questions about EYE DISCOMFORT:** a. During a typical day in the past month, **how often** did your eyes feel discomfort?   - NEVER [ ] 0 - RARELY [ ] 1 - SOMETIMES [ ] 2 - FREQUENTLY [ ] 3 - CONSTANTLY [ ] 4   b. When your eyes feel discomfort, **how intense** was this feeling of discomfort at the end of the day, within two hours of going to bed?   - NEVER HAVE IT [ ] 0 - NOT INTENSE AT ALL [ ] 1 - 2 - 3 - 4 - VERY INTENSE [ ] | **1.أسئلة حول الإحساس بعدم الارتياح في العين:**  **أ. خلال يوم عادي في الشهر الماضي، كم مرة شعرت بعدم الارتياح في عينك؟**   - أبدًا - نادرًا - أحياًنا - مرات عديدة - باستمرار   ب. عندما تشعر بعدم الراحة في عينيك، **ما مدى شدة هذا الشعور** بعدم الراحة في نهاية اليوم، خلال ساعتين من الذهاب إلى السرير؟   - لم أشعر به أبدًا - ليس شديد على الإطلاق - 2 - 3 - 4 - شديد جدًا 5 |
| **2. Questions about EYE DRYNESS:** a. During a typical day in the past month, **how often** did your eyes feel dry?   - NEVER [ ] 0 - RARELY [ ] 1 - SOMETIMES [ ] 2 - FREQUENTLY [ ] 3 - CONSTANTLY [ ] 4   b. When your eyes felt dry, **how intense** was this feeling of dryness at the end of the day, within two hours of going to bed?   - NEVER HAVE IT [ ] 0 - NOT INTENSE AT ALL [ ] 1 - 2 - 3 - 4 - VERY INTENSE [ ] 5 | **2.أسئلة حول جفاف العين:**  أ.خلال يومك عادي في الشهر الماضي، كم مرة شعرت بجفاف في عينيك؟   - أبدًا - نادرًا - أحيانًا - مرات عديدة - باستمرار   **ب.عندما تشعر بجفاف العين، ما مدى شدة هذا الشعور بالجفاف؟**   - أبدًا - نادرًا - احيانًا - مرات عديدة - دائمًا |
| **3. Questions about WATERY EYES:** a. During a typical day in the past month, **how often** did your eyes look or feel excessively watery?   - NEVER [ ] 0 - RARELY [ ] 1 - SOMETIMES [ ] 2 - FREQUENTLY [ ] 3 - CONSTANTLY [ ] 4 | **3.أسئلة حول العيون الدامعة:**  أ.خلال يوم عادي في الشهر الماضي، كم مرة بدت عيناك دامعة بشكل مفرط أو شعرت بها؟   - أبدًا - نادرًا - احيانًا - مرات عديدة - باستمرار |
| **1. Questions about EYE DISCOMFORT:** a. During a typical day in the past 2 weeks, **how often** did your eyes feel discomfort while wearing your contact lenses?   - 0 Never - 1 Rarely - 2 Sometimes - 3 Frequently - 4 Constantly   When your eyes felt discomfort with your contact lenses, **how intense** was this feeling of discomfort... b. At the end of your wearing time?   - 0 Never have it - 1 Not at All Intense - 2 - 3 - 4 - 5 Very Intense | **1. حول عدم الراحة في العينين:**  أ.خلال يوم عادي في الأسبوعين الماضيين، **كم مرة** شعرت بعدم الراحة في عينيك أثناء ارتداء العدسات اللاصقة؟   - أبدًا - نادرًا - أحيانًا - مرات عديدة - باستمرار   عندما شعرت بعدم الراحة في عينيك بسبب عدساتك اللاصقة، ما مدى شدة هذا الشعور بعدم الراحة؟   - لم أشعر به أبدًا - 1 ليس شديد على الإطلاق - 2 - 3 - 4 - شديد جدًا 5 |
| **3. Questions about CHANGEABLE, BLURRY VISION:** a. During a typical day in the past 2 weeks, **how often** did your vision change between clear and blurry or foggy while wearing your contact lenses?   - 0 Never - 1 Rarely - 2 Sometimes - 3 Frequently - 4 Constantly   When your vision was blurry, **how noticeable** was the changeable, blurry, or foggy vision... b. At the end of your wearing time?   - 0 Never have it - 1 Not at All Intense - 2 - 3 - 4 - 5 Very Intense | **.أسئلة حول الرؤية المتغيرة والضبابية**  ا.خلال يوم عادي في الأسبوعين الماضيين، كم مرة تغيرت رؤيتك بين الواضحة والضبابية أثناء ارتداء العدسات اللاصقة؟   - 0 أبدًا - 1 نادرًا - 2 أحيانًا - 3 مرات عديدة - دايمًا   عندما كانت رؤيتك ضبابية ، ما مدى وضوح الرؤية المتغيرة أو الضبابية أو مغيمه ...  b. في نهاية وقت ارتدائك؟   - 0 لم يكن لديك أبدا - 1 ليس مكثفا على الإطلاق - 2 - 3 - 4 - • 5 مكثف جدا |
| **4. Question about CLOSING YOUR EYES:** During a typical day in the past 2 weeks, **how often** did your eyes bother you so much that you wanted to close them?   - 0 Never - 1 Rarely - 2 Sometimes - 3 Frequently - 4 Constantly | **4.أسئلة حول اغلاق العين:**  خلال يوم عادي خلال الأسبوعين الماضيين، كم مرة أزعجتك عيناك لدرجة أنك أردت إغلاقهما؟   - أبدًا - نادرًا - أحيانًا - مرات عديدة - دائمًا |
| **5. Question about REMOVING YOUR LENSES:** How often during the past 2 weeks, did your eyes bother you so much while wearing your contact lenses that you felt as if you needed to stop whatever you were doing and take out your contact lenses?   1. Never 2. Less than once a week 3. Weekly 4. Several times a week 5. daily 6. Several times a day | **5.سؤال حول إزالة العدسات اللاصقة:**  كم مرة خلال الأسبوعين الماضيين، شعرت بإزعاج شديد في عينيك أثناء ارتداء العدسات اللاصقة لدرجة أنك شعرت وكأنك بحاجة إلى التوقف عن أي شيء كنت تفعله وإخراج العدسات اللاصقة؟   1. أبدًا 2. اقل من مرة في الأسبوع 3. اسبوعيًا 4. مرات عديدة في الأسبوع 5. يوميًا 6. مرات عديدة خلال اليوم |

◊ For each question below, circle one response from 0-4 in Column A.
➤ If your answer is 0 ('Never') in Column A, ➜ Move onto the next question.
➤ If your answer is 1-4 in Column A, ➜ Also circle one from 1-4 in Column B.

Please answer **all** questions without missing any.

| Column A | | | | | Column B | | | |  |
| --- | --- | --- | --- | --- | --- | --- | --- | --- | --- |
| Never | Occasionally | Sometimes | often | Always | Hardly bothered me | Bothered me a little | Bothered me | Bothered me very much |  |
| Grittiness (sensation of something in your eye)  During the past 7 days, did you experience the following symptoms? |  |  |  |  |  |  |  |  |  |
| Dry eyes |  |  |  |  |  |  |  |  |  |
| Sore eyes |  |  |  |  |  |  |  |  |  |
| Tired eyes |  |  |  |  |  |  |  |  |  |
| Heavy eyelids |  |  |  |  |  |  |  |  |  |
| Red eyes |  |  |  |  |  |  |  |  |  |

◊ لكل سؤال أدناه، اختر إجابة واحدة من 0 إلى 4 في العمود (أ)
➤ إذا كانت إجابتك 0 ("أبدًا") في العمود (أ) ➜ انتقل إلى السؤال التالي.
➤ إذا كانت إجابتك من 1 إلى 4 في العمود (أ) ➜ اختر أيضًا إجابة واحدة من 1 إلى 4 في العمود (ب)

**يرجى الإجابة على جميع الأسئلة دون تفويت أي منها.**

| عمود A | | | | | عمود B | | | |  |
| --- | --- | --- | --- | --- | --- | --- | --- | --- | --- |
| أبدًا  **خلال الأيام السبعة الماضية، هل عانيت من الأعراض التالية؟** | أحيانًا | بعض الأحيان | غالبًا | دائمًا | بالكاد أزعجني | أزعجني قليلًا | أزعجني | أزعجني جدًا |  |
| الشعور بجسم غريب (الإحساس بوجود شيء في العين) |  |  |  |  |  |  |  |  |  |
| جفاف العين |  |  |  |  |  |  |  |  |  |
| ألم العين |  |  |  |  |  |  |  |  |  |
| تعب العين |  |  |  |  |  |  |  |  |  |
| جفون ثقيلة |  |  |  |  |  |  |  |  |  |
| احمرار العين |  |  |  |  |  |  |  |  |  |
